# Predictive value of cervical shear wave elastography combined with cervical morphological parameters for preterm birth after cerclage in patients with cervical insufficiency

**DOI:** 10.3389/fmed.2026.1803636

**Published:** 2026-04-29

**Authors:** Minsui Cai, Yan Lu, Minhua Sheng, Juan Gao, Qi Cui

**Affiliations:** 1 Department of Ultrasound, Affiliated Hospital of Nantong University, Nantong, China; 2 Department of Radiology, Obstetrics and Gynecology Hospital of Fudan University, Shanghai, China; 3 Department of Ultrasound, the Affiliated People's Hospital of Jiangsu University, Zhenjiang, Jiangsu, China; 4 Department of Ultrasound, Wuxi Maternity and Child Health Care Hospital, Affiliated Women’s Hospital of Jiangnan University, Wuxi, Jiangsu, China

**Keywords:** anterior cervical angle, cervical length, shear wave elastography, spontaneous preterm birth, transvaginal cervical cerclage

## Abstract

**Purpose:**

This study aims to investigate the predictive value of shear-wave elastography (SWE) parameters, combined with cervical morphological parameters [cervical length (CL) and anterior cervical angle (ACA)] for spontaneous preterm birth (sPTB) in patients with cervical insufficiency following transvaginal cervical cerclage, thereby providing a reference for early clinical intervention.

**Patients and methods:**

Clinical data of 662 pregnant women diagnosed with cervical insufficiency who underwent vaginal cervical cerclage at Zhenjiang First People’s Hospital between January 1, 2018, and October 31, 2024, were retrospectively collected. Participants were divided into a preterm birth group (98 cases, <37 weeks) and a full-term group (564 cases, ≥37 weeks) based on gestational age at delivery. All patients underwent transvaginal ultrasound within 24 h before cerclage and during postoperative follow-up (19–36^+6^ weeks gestation) to measure CL, ACA, and SWE parameters, including Young’s modulus of the anterior external os (AE), anterior internal os (AI), peak Young’s modulus (PI), and mean Young’s modulus (PE). For predictive analysis, ultrasonographic parameters measured at 24–27^+6^ weeks were used. Univariate and multivariate logistic regression analyses were performed to identify independent risk factors for preterm birth. The predictive performance of individual indicators and their combination was evaluated using receiver operating characteristic (ROC) curve analysis.

**Results:**

CL and SWE parameters (AE, AI, PI, PE) were significantly lower in the preterm group than in the full-term group, while ACA was significantly higher (all *p* < 0.05). Multivariate logistic regression analysis showed that ACA > 111.55° was an independent risk factor for preterm birth after cerclage, while CL > 25.95 mm, AE > 8.845 kPa, AI > 14.01 kPa, PI > 16.47 kPa, and PE > 10.725 kPa were protective factors against preterm birth after cerclage (all *p* < 0.001). ROC curve analysis revealed that the combined detection model achieved an area under the curve (AUC) of 0.962, which was significantly higher than that of any single indicator (AUC range: 0.614–0.931). The sensitivity, specificity, and Youden index of the combined model were 96.7, 94.5%, and 0.912, respectively.

**Conclusion:**

SWE parameters combined with cervical morphological parameters are closely associated with sPTB after cerclage in patients with cervical insufficiency. The combination of these three indicators significantly improves predictive performance, providing reliable imaging-based evidence for precise pregnancy risk assessment and individualized intervention strategies.

**Plain language summary:**

Cervical shear-wave elastography parameters, in combination with cervical morphological parameters (cervical length and anterior cervical angle), can effectively predict the risk of spontaneous preterm birth in patients with cervical insufficiency after cerclage. The predictive model integrating these indicators demonstrates high accuracy and provides a reliable tool for clinical assessment, supporting individualized pregnancy monitoring and intervention strategies.

## Introduction

Preterm birth is a major contributor to perinatal morbidity and mortality, with approximately 15 million preterm infants born globally each year, of which 70–80% are due to spontaneous preterm birth (sPTB) (1). Cervical cerclage serves as a key intervention to prevent sPTB in high-risk pregnancies. Although it can prolong gestation to some extent, a subset of patients still experience preterm birth postoperatively, leading to adverse pregnancy outcomes (2). Therefore, identifying effective predictive markers for early recognition of individuals at persistent risk of preterm birth after cerclage is of significant clinical importance for personalized intervention and improved perinatal outcomes.

In clinical assessment, cervical morphological parameters are important indicators reflecting cervical structure and functional status. Among these, cervical length (CL) is the most classic and widely used morphological parameter, traditionally employed for predicting preterm birth risk (3). However, after cerclage, the accuracy of relying solely on CL measurement is limited due to the alteration of cervical anatomy by the suture (4). In recent years, the anterior cervical angle (ACA), an emerging morphological indicator, has gained attention. It reflects morphological changes and load-bearing status of the cervix under intrauterine pressure and is considered associated with mechanical stress on the cervix and the risk of preterm birth (5). Concurrently, cervical shear-wave elastography (SWE) technology provides a novel biomechanical perspective for assessing cervical function. It objectively reflects cervical softening by quantifying tissue stiffness (elastic modulus), and cervical softening has been confirmed to be closely related to the occurrence of preterm birth (6).

Currently, although the individual application of these indicators has demonstrated certain predictive value, systematic research on the combined use of SWE parameters and cervical morphological parameters (CL and ACA) for multidimensional assessment of preterm birth risk after cerclage remains insufficient. The synergistic effects and comprehensive predictive efficacy of these indicators require further clarification. Therefore, this study aims to investigate the predictive value of cervical SWE combined with cervical morphological parameters for preterm birth in patients with cervical insufficiency after cerclage, with the goal of providing more reliable evidence for early clinical risk stratification and intervention decisions, thereby optimizing perinatal management and improving maternal and neonatal outcomes.

## Materials and methods

### Design and setting

Clinical data were retrospectively collected from 662 pregnant women diagnosed with cervical insufficiency who underwent vaginal cervical cerclage at Zhenjiang First People’s Hospital between January 1, 2018, and October 31, 2024. According to the gestational age at delivery, participants were divided into a preterm birth group (98 cases, <37 weeks) and a full-term group (564 cases, ≥37 weeks). Based on the gestational age at the time of postoperative examination, the patients were further stratified into four subgroups: 19–23^+6^ weeks, 24–27^+6^ weeks, 28–33^+6^ weeks, and 34–36^+6^ weeks. This study was approved by the Ethics Committee of Zhenjiang First People’s Hospital [Approval No. (2025) KYW001-23]. According to the standardized clinical management protocol for cervical cerclage at our institution, all patients who underwent cervical cerclage were required to undergo at least one transvaginal ultrasound examination during the postoperative follow-up period (19–36^+6^ weeks) to assess cervical morphology and functional status.

Diagnostic Criteria for Cervical Insufficiency: According to the American College of Obstetricians and Gynecologists (7), cervical insufficiency can be diagnosed if any of the following criteria are met: (1) History-indicated diagnosis: A history of at least one prior painless cervical dilation resulting in delivery of a live fetus in the mid-to-late second trimester or early third trimester, in the absence of uterine contractions or signs of threatened abortion or preterm labor, and after excluding other pathological factors such as infection, bleeding, and placental abruption. (2) Physical examination-indicated diagnosis: Painless cervical dilation detected in the second trimester or early third trimester, accompanied by cervical softening, effacement, or dilation, with visible herniation of the fetal membranes into the cervical canal or vagina. (3) Ultrasound-indicated diagnosis: Cervical length ≤25 mm measured by transvaginal ultrasound before 24 weeks of gestation, with progressive cervical dilation.

Inclusion criteria were as follows: (1) age ≥18 years; (2) singleton pregnancy without significant cervical inflammation; (3) Complete clinical data available, including pre-cerclage ultrasound measurements (CL, ACA, and SWE parameters) obtained within 24 h before surgery, at least one set of post-cerclage ultrasound measurements during follow-up (19–36^+6^ weeks), documented pregnancy outcome (gestational age at delivery, delivery mode, neonatal birth weight), complete operative records, and postpartum follow-up data. Exclusion criteria included: (1) termination of pregnancy due to fetal factors, such as fetal distress or fetal malformations; (2) severe gestational comorbidities or complications; (3) uterine malformations, among other relevant conditions; (4) Multiple pregnancy.

Indications for cerclage: According to standard clinical practice, the indications for cervical cerclage were classified into three categories: (1) History-indicated cerclage was performed in patients with a prior painless mid-trimester pregnancy loss (16–28 weeks) attributed to cervical insufficiency, or a history of cervical conization/LEEP procedure with residual cervical length <25 mm; (2) Ultrasound-indicated cerclage was offered to patients with a sonographic cervical length ≤25 mm with or without funneling of the internal os in the current pregnancy, regardless of prior obstetric history; (3) Physical examination-indicated cerclage was performed in patients presenting with painless cervical dilation ≥1–2 cm with visible or palpable amniotic sac during the second trimester (14–27 weeks), in the absence of regular uterine contractions or evidence of infection.

### Surgical method

The McDonald cerclage procedure was performed. The patient was placed in the lithotomy position. Following the administration of spinal anesthesia, routine disinfection of the vulva and vagina was carried out, and sterile drapes were applied. The bladder was emptied using a catheter. The cervix was exposed with vaginal retractors, grasped with Allis forceps, and gently pulled downward and outward. A double-stranded No. 10 silk suture was used to place a purse-string suture in the cervical stroma as high as possible at the level of the cervicovaginal junction, close to the internal cervical os. The suture was inserted sequentially at the 1 o’clock position, exiting near the 11 o’clock position; inserted at the 10 o’clock position, exiting at the 8 o’clock position; inserted at the 7 o’clock position, exiting at the 5 o’clock position; and inserted at the 4 o’clock position, exiting at the 2 o’clock position. Finally, the suture encircling the cervix was tightened to close the internal os and tied at the anterior vaginal fornix, leaving suture tails of 2 cm in length. It was confirmed that a No. 4 cervical dilator could pass through the internal cervical os. After checking the cervix for any bleeding or exudation, the vagina was re-disinfected and an indwelling urinary catheter was placed, concluding the procedure. In case of cervical bleeding, two gauze pads soaked with iodophor could be applied for compression and removed postoperatively.

### Ultrasonographic examination protocol

**Figure 1:**
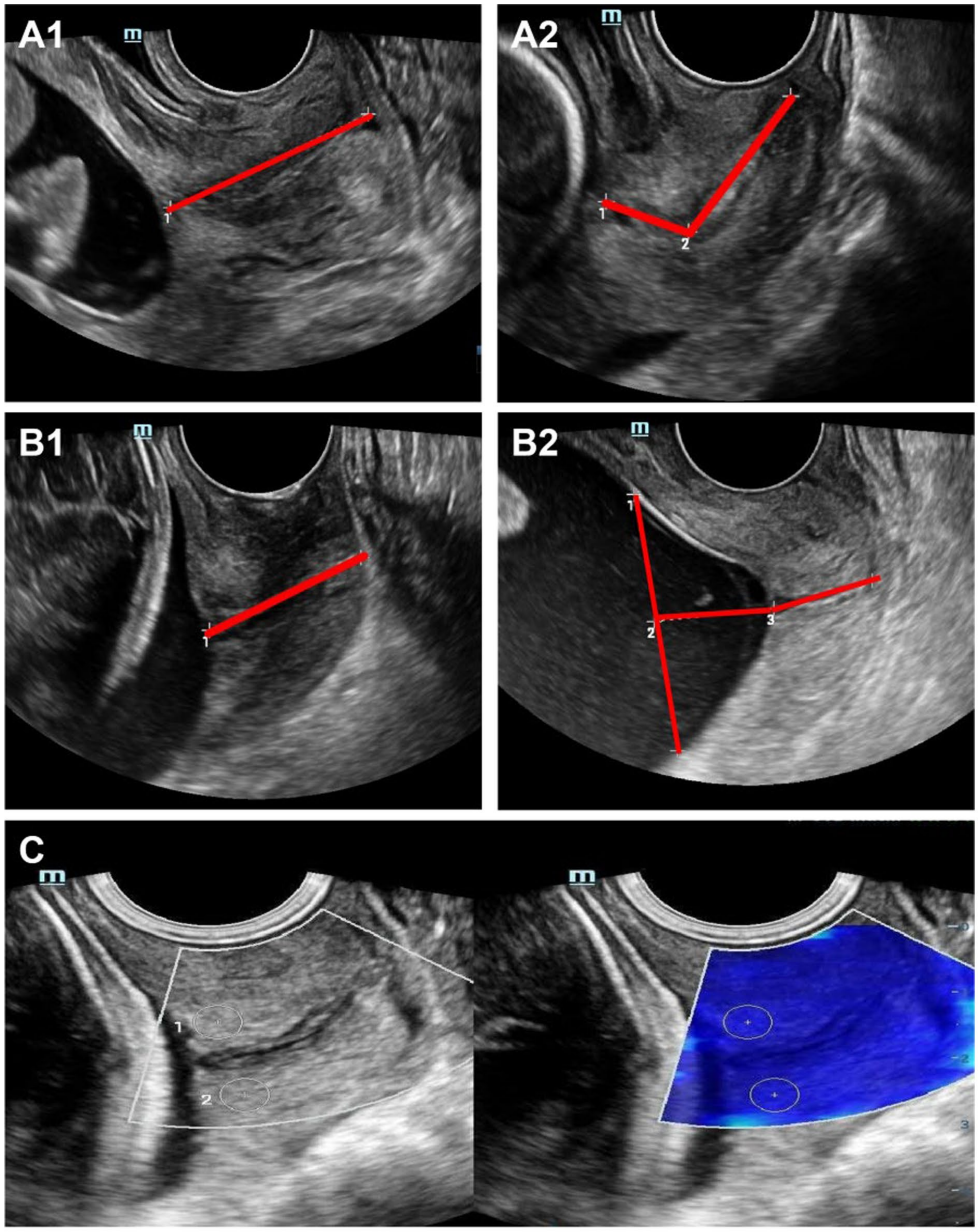
Schematic diagram of ultrasound examination. **(A)** Transvaginal ultrasound measurement of CL. **(A1)** Straight one-step method. **(A2)** Straight two-step method. **(B)** Measurement of the ACA of the internal os. **(B1)** Closed internal cervical os. **(B2)** Open internal cervical os. **(C)** Measurement of cervical SWE.

Examinations were performed using a Mindray Resona R9 Pro/Eagus R9s ultrasound system equipped with SWE software and a DE103WU transvaginal probe (frequency range: 3–11 MHz). The measurement procedures were conducted as follows: (1) CL measurement: After instructing the participants to empty their bladders, they were placed in the lithotomy position. The probe was gently inserted into the anterior vaginal fornix without excessive pressure. On a standard sagittal view, the image was magnified to >75% of the screen to clearly visualize the entire cervix from the internal to the external os. Measurement methods were chosen based on cervical morphology: for a straight cervix, a one-step linear method was used to directly measure the linear distance between the internal and external os; for a curved cervix, a two-step linear method was applied, measuring the distances from the internal os to the curvature point and from the curvature point to the external os, with the two values summed. Three consecutive measurements were taken for each case, and the minimum value was recorded. Care was taken to avoid cervical compression by a full bladder and to exclude cervical funneling artifacts caused by probe pressure or uterine contractions. If true cervical funneling was present, the distance from the apex of the funnel to the external os was measured (Figure 1A). (2) ACA measurement: When the internal cervical os is closed, a straight line (L1) is drawn from the internal os to the external cervical os, and another straight line (L2) is drawn from the internal os along the lower uterine segment of the anterior wall. The angle formed between L1 and L2 is defined as the ACA. In cases where the internal cervical os is partially dilated, L1 is drawn from the point of cervical closure to the external os, and L2 is drawn along the lower anterior uterine segment. The angle between these two lines is then measured as the ACA. Each measurement is performed three times, and the average value is used for the final analysis. The ACA was measured on a sagittal view by defining the line connecting the centers of the internal and external os as L1, and drawing a line parallel to the serosal surface of the lower anterior uterine wall through the internal os as L2. The angle between L1 and L2 was defined as the ACA (Figure 1B). (3) SWE measurement: Following morphometric assessments, SWE mode was activated. During acquisition, motion stability index (M-STB) and reliability index (RLBindex) were monitored in real time. Images were frozen only when motion artifacts were low (M-STB ≥ 4 stars) and the reliable region percentage was high (RLBindex > 95%). Circular regions of interest (ROIs, approximately 2 mm in diameter) were placed on the anterior lip of the external os and the anterior lip of the internal os, respectively. The system automatically acquired shear-wave velocity (*Vs*), and Young’s modulus (E) was calculated using the formula E = 3*ρ*Vs^2^, where tissue density (ρ) was set to 1,000 kg/m^3^. Results were recorded and displayed in kilopascals (kPa) (Figure 1C).

### Data collection

The collected data included: (1) Baseline clinical information such as age, pre-pregnancy body mass index, gestational age at the time of cerclage, and primiparity; (2) ultrasonographic findings: In the two patient groups, CL, ACA, and SWE parameters [including the Young’s modulus value of the external os of the anterior cervical lip (AE), the Young’s modulus value of the internal os of the anterior cervical lip (AI), the peak Young’s modulus value (PI), and the mean Young’s modulus value (PE)] were measured within 24 h before cervical cerclage and during postoperative follow-up at different gestational weeks (19–23^+6^ weeks, 24–27^+6^ weeks, 28–33^+6^ weeks, and 34–36^+6^ weeks); (3) analytical content: multivariate analysis was performed to explore risk factors for sPTB, and receiver operating characteristic curves were plotted to assess the predictive performance of individual and combined ultrasound indicators for sPTB, with calculations of the area under the curve, optimal predictive thresholds, and corresponding sensitivity, specificity, and accuracy.

### Statistical analysis

Statistical analysis was performed using SPSS version 22.0. Normally distributed quantitative data were expressed as mean ± standard deviation, and intergroup comparisons were conducted using one-way analysis of variance. Non-normally distributed quantitative data were presented as median and interquartile range, with intergroup comparisons performed using the Kruskal–Wallis H test. Qualitative data were expressed as counts and percentages, and intergroup comparisons were conducted using Pearson’s chi-square test or Fisher’s exact test. Pre-cerclage ultrasonographic parameters were compared between the preterm and full-term groups to assess baseline differences. To standardize the timing of postoperative cervical assessment and minimize confounding due to varying gestational ages at measurement, ultrasonographic parameters measured at 24–27^+6^ weeks were selected as independent variables in logistic regression and ROC analyses for predicting preterm birth. This gestational interval represented the predominant postoperative follow-up window in our cohort (accounting for 64.18% of the full-term group and 62.24% of the preterm group) and coincides with a critical period for cervical remodeling and preterm birth risk assessment. Using measurements from this relatively narrow gestational window enhances the clinical comparability and interpretability of the predictive model. A *p*-value < 0.05 was considered statistically significant.

## Results

### Baseline characteristics

A total of 662 pregnant women were included in this study, among whom 98 (14.80%) were in the preterm birth group. The distribution of gestational age at delivery in the preterm group was as follows: extreme preterm (<28 weeks) in 3 cases (3.06%), very preterm (28–31^+6^ weeks) in 11 cases (11.22%), moderate preterm (32–33^+6^ weeks) in 13 cases (13.27%), and late preterm (34–36^+6^ weeks) in 71 cases (72.45%). Baseline characteristics were compared between the two groups, showing no statistically significant differences in age, pre-pregnancy BMI, cerclage indication, or time of ultrasound examination after cerclage (*p* > 0.05). However, there was a statistically significant difference in gestational age at delivery and gestational age at cerclage (*p* < 0.05). Furthermore, the proportions of previous abortion history and preterm birth history were significantly higher in the preterm group compared with the term group (*p* < 0.05). Details are presented in Table 1.

**Table 1:** Comparison of baseline characteristics between the two groups.

Indicators	Full-term group *n* = 564	Preterm birth group *n* = 98	*Z*/*t*/*χ*^2^	*P*
Age/years	31.74 ± 4.18	30.83 ± 4.65	1.955	0.051
Pre-pregnancy BMI/kg·m^−2^	21.74 ± 2.05	22.12 ± 2.41	1.661	0.097
Cerclage indication			3.680	0.159
History-indicated	257 (45.57)	51 (52.40)		
Ultrasound-indicated	273 (48.40)	38 (38.78)		
Physical examination-indicated	34 (6.03)	9 (9.18)		
Gestational age at delivery	39.03 ± 0.51	33.62 ± 0.74	89.901	< 0.001
Gestational age at cerclage			20.417	< 0.001
12 ~ < 15 weeks	293 (51.95)	31 (31.63)		
15 ~ 16 weeks	144 (25.53)	46 (46.94)		
> 16 weeks	127 (22.52)	21 (21.43)		
Time of ultrasound examination after cerclage			5.464	0.141
GA 19–23^+6^ weeks	85 (15.07)	23 (23.47)		
GA 24 ~ 27^+6^ weeks	362 (64.18)	61 (62.24)		
GA 28 ~ 33^+6^ weeks	79 (14.01)	9 (9.18)		
GA 34 ~ 36^+6^ weeks	38 (6.74)	5 (5.10)		
Primipara			3.017	0.082
Yes	482 (85.46)	77 (78.57)		
No	82 (14.54)	21 (21.43)		
History of abortion	75 (13.30)	11 (11.22)	5.887	0.015
History of preterm birth	12 (2.13)	13 (13.27)	25.519	< 0.001

GA, gestational age.

### Ultrasonographic findings before cerclage

The preterm birth group showed a shorter CL compared with the term group (25.88 ± 9.79 mm vs. 30.07 ± 11.45 mm, *p* < 0.05). The ACA was larger in the preterm group than in the term group (120.19 ± 13.66° vs. 103.20 ± 15.72°, *p* < 0.05). Furthermore, the Young’s modulus values at the AE, AI, PI, and PE were all significantly lower in the preterm group than in the term group (all *p* < 0.05). Detailed data are presented in Table 2.

**Table 2:** Comparison of pre-cerclage ultrasonographic parameters between the two groups.

Indicators	Full-term group *n* = 564	Preterm birth group *n* = 98	*t*	*P*
CL/mm	30.07 ± 11.45	25.88 ± 9.79	3.001	0.003
ACA/°	103.20 ± 15.72	120.19 ± 13.66	−13.682	< 0.001
SWE/kPa				
AE	10.03 ± 2.36	7.14 ± 2.18	2.849	0.005
AI	17.35 ± 6.24	11.54 ± 3.19	11.638	< 0.001
PI	22.27 ± 4.83	11.87 ± 3.04	17.214	< 0.001
PE	13.94 ± 4.87	8.20 ± 5.01	6.605	< 0.001

Correlation of CL, ACA, and SWE with gestational age.

**Figure 2:**
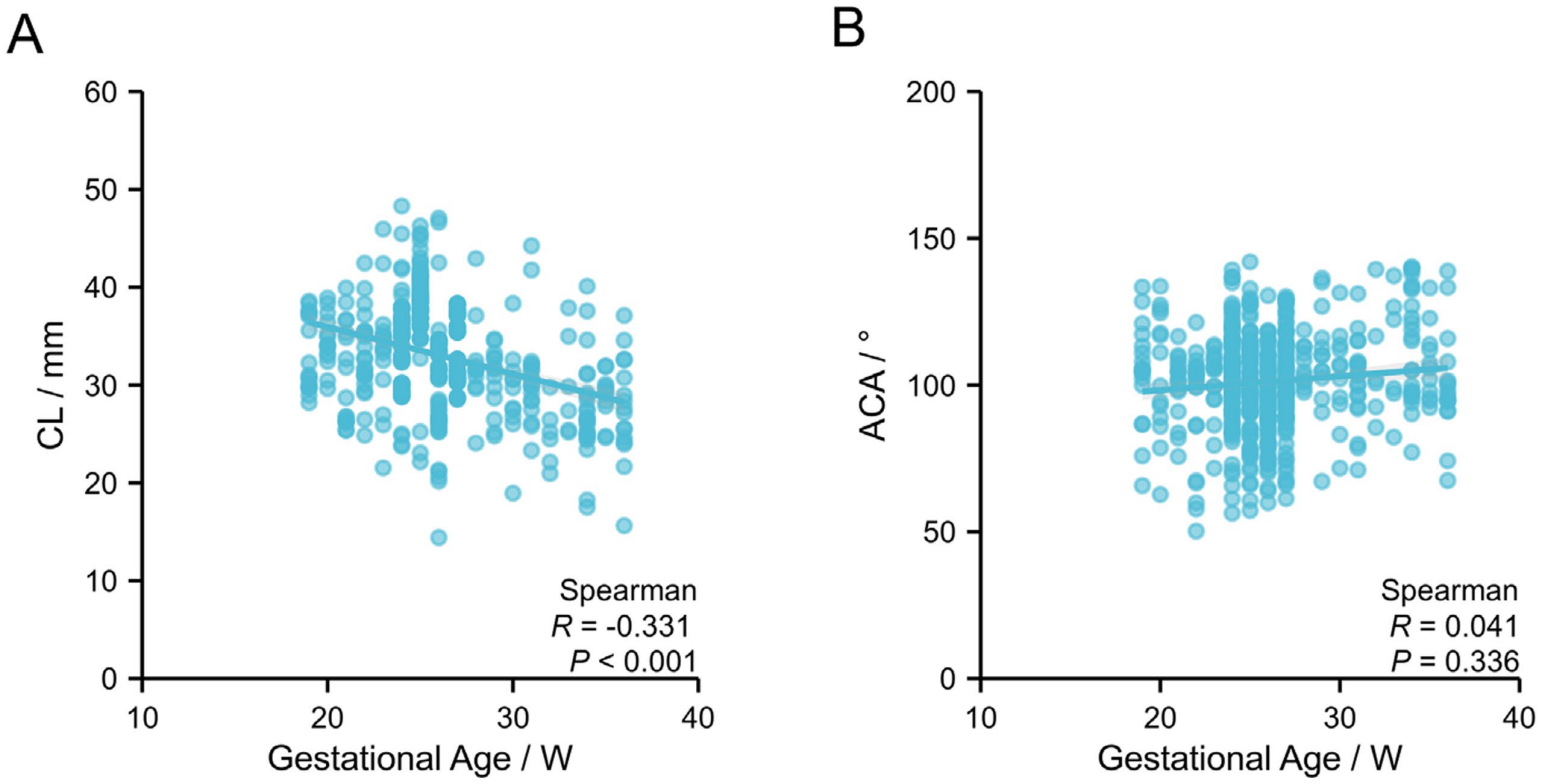
Correlation of CL and ACA with gestational age. **(A)** Correlation between CL and gestational age. **(B)** Correlation between ACA and gestational age.

**Figure 3:**
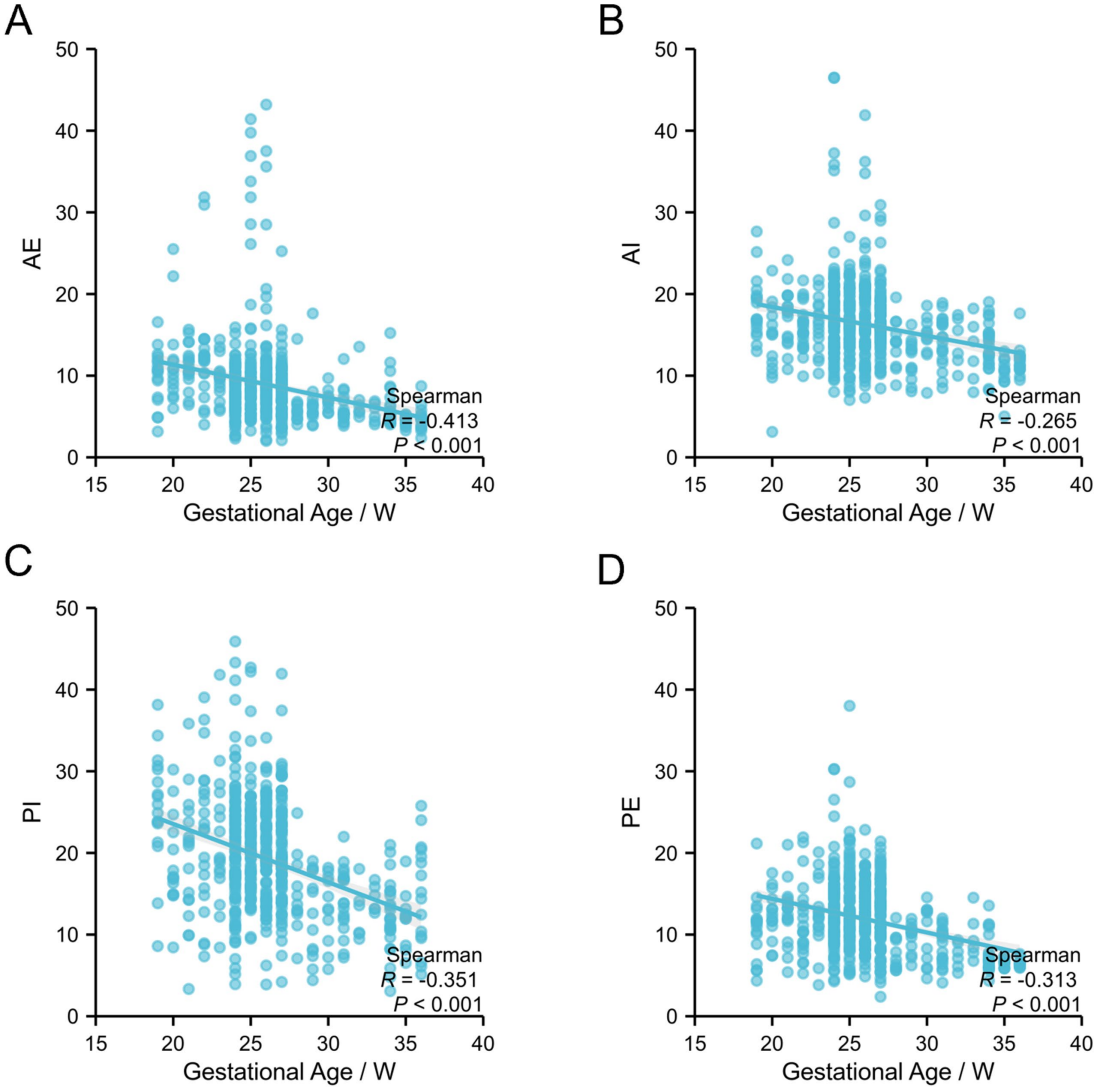
Relationship between SWE parameters at different cervical locations and gestational age. Correlation of shear-wave elastography parameters with gestational age. **(A)** AE versus gestational age. **(B)** AI versus gestational age. **(C)** PI versus gestational age. **(D)** PE versus gestational age.

To investigate the correlation of CL, ACA, and SWE parameters with gestational age, data from the term group were analyzed. The results showed that CL gradually decreased with advancing gestational age, and Spearman correlation analysis indicated a significant negative correlation between CL and gestational age (*R* = −0.331, *p* < 0.001). No significant correlation was observed between ACA and gestational age (*p* > 0.05), suggesting that ACA remained relatively stable throughout gestation (Table 3; Figure 2). In addition, all SWE parameters—AE, AI, PI, and PE—were negatively correlated with gestational age, showing a progressive decline as gestational age increased (Table 4; Figure 3).

**Table 3:** Relationship between ultrasonographic parameters and gestational age.

Indicators	19 ~ 23^+6^ weeks	24 ~ 27^+6^ weeks	28 ~ 33^+6^ weeks	34 ~ 36^+6^ weeks	*F*	*P*
*n*	85	362	79	38		
CL/mm	29.74 ± 9.35	29.13 ± 10.62	28.17 ± 8.94	27.31 ± 9.09	54.199	< 0.001
ACA/°	104.56 ± 6.39	100.27 ± 9.30	102.49 ± 11.30	94.82 ± 10.25	134.071	< 0.001
SWE/kPa						
AE	10.26 ± 3.07	8.04 ± 2.83	5.94 ± 1.17	4.72 ± 0.85	32.216	< 0.001
AI	16.88 ± 4.25	16.59 ± 3.72	14.03 ± 2.85	11.64 ± 2.27	51.640	< 0.001
PI	21.51 ± 7.48	19.96 ± 6.25	13.62 ± 3.87	12.53 ± 4.16	40.072	< 0.001
PE	13.07 ± 3.88	12.85 ± 4.09	8.42 ± 2.65	6.49 ± 0.58	54.217	< 0.001

**Table 4:** Univariate analysis of ultrasonographic parameters.

Indicators	sPTB		*Z*	*P*
	No	Yes		
CL/mm	29.44 (22.44, 36.45)	24.49 (18.15, 27.99)	−5.162	< 0.001
ACA/°	99.28 (93.24, 106.92)	119.15 (113.66, 129.31)	−10.763	< 0.001
SWE/kPa				
AE	8.22 (6.04, 10.01)	7.24 (5.68, 8.46)	−2.852	0.004
AI	16.34 (13.95, 19.19)	11.08 (8.60, 13.59)	−9.103	< 0.001
PI	19.81 (15.74, 24.46)	11.38 (8.89, 13.44)	−10.119	< 0.001
PE	12.72 (9.92, 15.47)	8.22 (3.85, 10.72)	−6.830	< 0.001

### Prediction of sPTB using ultrasonographic parameters

**Figure 4:**
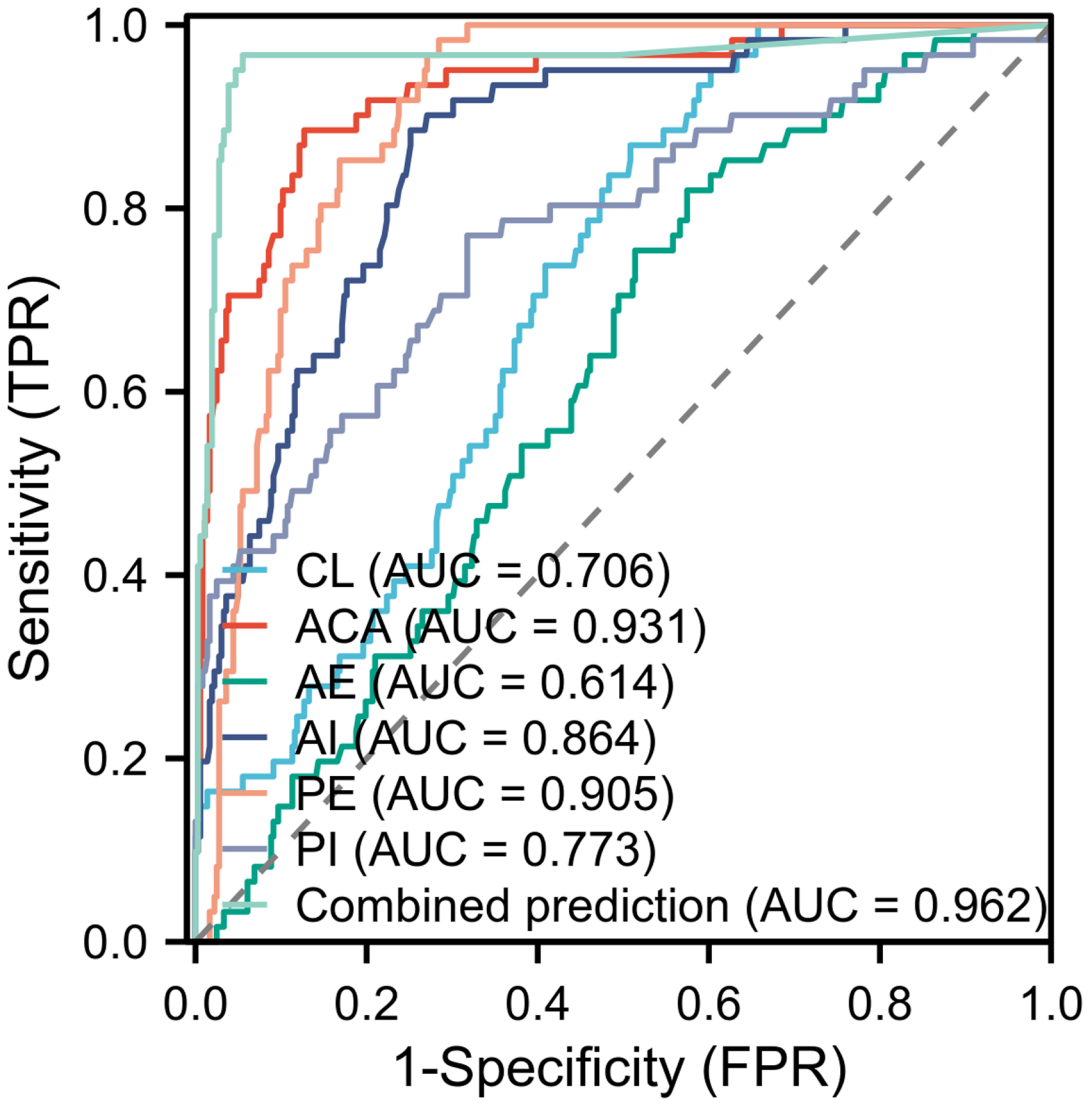
ROC curve.

Ultrasonographic parameters measured at 24 to 27^+6^ weeks of gestation (the predominant post-cerclage follow-up interval, accounting for 64.18% of the full-term group and 62.24% of the preterm group) were selected for predictive analysis. Univariate analysis showed that CL, ACA, and SWE parameters differed significantly between the two groups (*p* < 0.05), as presented in Table 4. Subsequently, binary logistic regression analysis was performed using the statistically significant indicators from univariate analysis as independent variables and the occurrence of preterm birth as the dependent variable (Table 5). The results indicated that CL > 25.95 mm (*OR* = 0.156, 95% *CI*: 0.072–0.337), AE > 8.845 kPa (*OR* = 0.297, 95% *CI*: 0.150–0.590), AI > 14.01 kPa (*OR* = 0.044, 95% *CI*: 0.019–0.099), PI > 16.47 kPa (*OR* = 0.007, 95% *CI*: 0.001–0.048), and PE > 10.725 kPa (*OR* = 0.139, 95% *CI*: 0.073–0.262) were protective factors against preterm birth, while ACA > 111.55° (*OR* = 1.694, 95% *CI*: 1.283–2.249) was an independent risk factor for preterm birth after cerclage (all *p* < 0.001), with all variance inflation factors (VIF) < 5. ROC curve analysis was then conducted to evaluate the predictive performance of each parameter for preterm birth. The combined detection model achieved an AUC of 0.962, which was significantly higher than that of any single parameter (AUC range: 0.614–0.931). The sensitivity, specificity, and Youden index of the combined model were 96.7, 94.5%, and 0.912, respectively, indicating good predictive value (Table 6; Figure 4).

**Table 5:** Multivariate logistic regression analysis of sPTB.

Indicators	*B*	*SE*	*Z*	*P*	*OR* (95% *CI*)	VIF
CL > 25.95	−1.858	0.394	−4.720	<0.001	0.156 (0.072 ~ 0.337)	1.055
ACA > 111.55	0.527	0.143	3.690	<0.001	1.694 (1.283 ~ 2.249)	1.212
AE > 8.845	−1.214	0.350	−3.471	<0.001	0.297 (0.150 ~ 0.590)	1.025
AI > 14.01	−3.134	0.420	−7.470	<0.001	0.044 (0.019 ~ 0.099)	1.129
PI > 16.47	−5.016	1.015	−4.942	<0.001	0.007 (0.001 ~ 0.048)	1.151
PE > 10.725	−1.976	0.325	−6.084	<0.001	0.139 (0.073 ~ 0.262)	1.066

**Table 6:** ROC curve performance analysis.

Indicators	AUC	Cut off	Sensitivity / %	Specificity / %	Youden index	95% *CI*
CL	0.706	25.95	0.869	0.492	0.361	0.648 ~ 0.765
ACA	0.931	111.55	0.885	0.873	0.758	0.895 ~ 0.966
AE	0.614	8.845	0.820	0.425	0.245	0.547 ~ 0.682
AI	0.864	14.01	0.885	0.749	0.634	0.819 ~ 0.909
PI	0.905	16.47	0.984	0.715	0.699	0.875 ~ 0.934
PE	0.773	10.725	0.770	0.682	0.453	0.702 ~ 0.844
Combined prediction	0.962	0.045	0.967	0.945	0.912	0.927 ~ 0.996

## Discussion

This study systematically analyzed clinical data and ultrasonographic findings from 662 patients who underwent cerclage for cervical insufficiency. It specifically evaluated the value of SWE parameters and cervical morphological parameters in predicting spontaneous preterm birth following the procedure. The results demonstrate that the combined assessment of these parameters provides excellent predictive performance, offering a valuable reference for the precise clinical evaluation of pregnancy risk.

In this study, the rates of previous abortion and preterm birth were significantly higher in the preterm group than in the term group (*p* < 0.05), which is consistent with previous findings (8). Women with a history of adverse pregnancy outcomes may have subclinical cervical injury or congenital structural defects; even after cerclage, the mechanical support function of the cervix may remain weaker than that of women without such a history, thereby increasing the risk of preterm birth (9). oreover, a significant difference in gestational age at cerclage was observed between the two groups, with patients who received cerclage at 15–16 weeks showing a relatively higher proportion of preterm birth. This observation differs slightly from the findings of Berghella et al. (10), who proposed 12–14 weeks as the optimal timing for cerclage. In their view, performing the procedure too early may increase technical difficulty due to inadequate cervical exposure, whereas delayed intervention might reduce its efficacy as the cervix could have already undergone irreversible dilatation. In the current cohort, the increased preterm risk associated with cerclage at 15–16 weeks may be attributable to early cervical softening already present in some patients during this period, suggesting that surgical intervention may not fully halt the progressive decline in cervical competence. This finding underscores the importance of a dynamic assessment based on cervical morphological and functional indicators rather than relying solely on gestational age to determine the timing of cerclage. Notably, among the 98 preterm births in our cohort, 72.45% were late preterm (34–36^+6^ weeks), while only 3.06% were extreme preterm (<28 weeks). This distribution suggests that although cervical cerclage may not completely prevent preterm birth in all patients, it may help prolong pregnancy to later gestational ages with better neonatal outcomes in a substantial proportion of cases. The heterogeneity in preterm birth severity also underscores the need for individualized risk stratification and targeted interventions based on comprehensive cervical assessment.

CL is a classical indicator for assessing cervical competence, and its shortening is widely recognized as an important early warning sign of preterm birth (11). In this study, CL was significantly shorter in the preterm group than in the term group (*p* = 0.003). ROC curve analysis showed that CL yielded an AUC of 0.706 for predicting preterm birth, with a cut-off value of 29.95 mm, and sensitivity and specificity of 86.9 and 49.2%, respectively. These findings are consistent with the multicenter study by Salomon et al. (12), which reported an increased risk of preterm birth after cerclage when CL was <30 mm, with an AUC ranging from 0.68 to 0.72 and specificity limited to 45–50%. Together, these results suggest that CL has some predictive value for preterm birth after cerclage; however, its low specificity makes it inadequate as a standalone screening tool for precise risk stratification. Further analysis of the relationship between CL and gestational age revealed that in the term group, CL gradually decreased with advancing gestational age (R = −0.331, *p* < 0.001). This pattern aligns with the physiological process of cervical maturation during pregnancy, in which the cervix progressively shortens and softens in preparation for parturition (13, 14). In contrast, cervical shortening was more pronounced in the preterm group, indicating an abnormally accelerated cervical maturation under pathological conditions. This observation provides a plausible explanation for the increased risk of preterm birth in this population.

The ACA reflects the angular relationship between the cervix and the lower uterine segment, and its widening may indicate relaxation and progressive dilation of the internal cervical os (15). In this study, the ACA was significantly larger in the preterm group than in the term group (*p* < 0.001). ROC curve analysis yielded an AUC of 0.931 for ACA, with sensitivity and specificity of 88.5 and 87.3%, respectively, demonstrating a significantly better predictive performance than CL. These findings are highly consistent with the results reported by Chen et al. (16), in which an ACA > 110° was associated with a significantly increased risk of preterm birth after cerclage. The cut-off value of ACA identified in the present study (111.55°) is similar to the 110° reported by Chen et al., further supporting the reliability of ACA as a predictor of preterm birth following cerclage. Multivariate logistic regression analysis revealed that ACA was the only parameter with an *OR* > 1, indicating that a wider anterior cervical angle (ACA > 111.55°) significantly elevates the risk of preterm birth (*OR* = 1.694, 95% *CI*: 1.283–2.249), while all other parameters (CL and SWE metrics) demonstrated protective effects. The underlying mechanism may be that an enlarged ACA alters the distribution of pressure from the fetus and amniotic sac on the internal cervical os, thereby reducing cervical support and increasing the likelihood of preterm birth (17). It is worth noting that although ACA generally tended to decrease with advancing gestational age, correlation analysis did not show a statistically significant negative association, which may be attributed to individual variations in cervical morphology and alterations in the biomechanical structure of the cervix after cerclage.

SWE quantitatively assesses cervical tissue stiffness by calculating Young’s modulus based on shear-wave velocity, providing an objective measurement that may reflect early cervical dysfunction (18). In the present study, SWE-derived parameters—including the Young’s modulus at the AE, AI, PI, and PE—were significantly lower in the preterm group than in the term group (all *p* < 0.001). Among these parameters, PI achieved an AUC of 0.905 for predicting preterm birth, with a sensitivity as high as 98.4%, while AI yielded an AUC of 0.864 with a specificity of 74.9%. Recent studies have similarly shown that SWE parameters focusing on the internal os/posterior lip, either alone or in combination with CL, generally achieve AUCs ranging from 0.81 to 0.89 in populations with cervical insufficiency, further supporting the predictive utility of SWE (18, 19). Moreover, in the term group, all SWE parameters demonstrated a significant downward trend with advancing gestational age (*p* < 0.001), in line with the physiological softening of cervical tissue during cervical maturation. Histological studies in both animal and human tissues have confirmed that increased collagen degradation during cervical maturation leads to reduced tissue stiffness. The more pronounced decrease in cervical tissue stiffness observed in the preterm group suggests an abnormally accelerated maturation process, which aligns with and corroborates the conclusions drawn from the CL analysis (20, 21). This finding is consistent with the protective role of higher cervical stiffness (as reflected by higher AE, AI, PI, and PE values) identified in the multivariate analysis, further supporting that maintaining cervical tissue integrity is crucial for preventing preterm birth after cerclage.

Multivariate logistic regression analysis indicated that ACA was an independent risk factor, while CL and all SWE parameters (AE, AI, PI, PE) were independent protective factors for preterm birth after cerclage. To standardize the timing of postoperative cervical assessment, ultrasonographic parameters measured at 24–27^+6^ weeks were selected for predictive analysis. This gestational interval holds important clinical significance: it represents a critical phase of cervical remodeling, offers good clinical operability with patients in the mid-trimester, and had the highest follow-up data coverage in our cohort (64.18% in the term group and 62.24% in the preterm group). The combined predictive model achieved an AUC of 0.962, with a sensitivity of 96.7% and specificity of 94.5%, significantly higher than any single indicator. This result outperforms existing studies: Angelopoulou et al. (8) showed a pooled AUC of 0.82 (95% confidence interval: 0.78–0.85) for cervical elastography alone in predicting preterm birth; Sun et al. (22) reported an overall AUC of 0.87 (95% *CI*: 0.72–0.95) for cervical elastography, with the SWE subgroup demonstrating a pooled sensitivity of 88% and specificity of 71%. The superior predictive performance of the combined model in this study may be attributed to two main aspects. First, the inclusion of ACA, a key morphological indicator, compensated for the limitations of relying solely on a single morphological (CL only) or functional (SWE only) parameter, enabling a multidimensional integrated assessment of cervical morphology and function. Second, the SWE parameters encompassed measurements of both the anterior internal os and anterior external os, providing a more comprehensive reflection of overall cervical elasticity. Moreover, this study strictly controlled for confounding factors such as infection and uterine anomalies, thereby minimizing interference with predictive performance.

Based on the high-risk cervical conditions identified in this study, including shortened cervical length, widened anterior cervical angle, and reduced cervical tissue stiffness, the following interventions may be considered to reduce preterm birth risk. First, enhanced perinatal surveillance: more frequent ultrasound follow-up and patient education for those with persistently shortened cervical length or significantly increased anterior cervical angle. Second, progesterone administration may be considered for patients with significantly reduced cervical stiffness indicated by SWE parameters (23). Third, for patients with progressive cervical shortening accompanied by funneling, repeat or rescue cerclage may be considered by an experienced physician after strictly excluding infection (24). Finally, for patients at extremely high risk, early communication with the neonatology department and timely administration of antenatal corticosteroids are recommended.

### Limatations and future directions

This study has several limitations. First, as a single-center retrospective cohort investigation, potential selection bias may exist despite the relatively large sample size. Second, the ultrasonographic examinations and parameter measurements were operator-dependent, which might introduce measurement variability. Third, the combined predictive model was developed and tested on the same dataset without internal or external validation; therefore, the apparent predictive performance (AUC = 0.962) may be overoptimistic and the risk of overfitting cannot be fully excluded. Although the events-per-variable ratio (EPV = 16.3) exceeded the recommended minimum of 10, suggesting a relatively low risk of severe overfitting, this does not substitute for formal validation. To address these limitations, future multicenter prospective studies with independent external cohorts are needed to validate our findings. Standardized protocols for ultrasound examinations should be established to minimize operator-dependent variability, and integrating additional biomarkers may further improve predictive performance for clinical application.

## Conclusion

In summary, cervical morphological and SWE parameters are closely associated with spontaneous preterm birth after cerclage: a wider anterior cervical angle increases risk, while longer cervical length and higher cervical tissue stiffness (as reflected by SWE parameters) confer protective effects. The combined use of these indicators significantly enhances predictive accuracy, offering a reliable basis for early identification of high-risk populations and timely clinical intervention. This approach holds important clinical implications for improving maternal and neonatal outcomes.

## Data Availability

The raw data supporting the conclusions of this article will be made available by the authors, without undue reservation.
